# Effects of Moral Elevation on Children’s Implicit and Explicit Prosociality: Evidence from Behavioral and Physiological Responses

**DOI:** 10.3390/bs15091246

**Published:** 2025-09-12

**Authors:** Qin Wang, Xia Zhou, Lei Xun

**Affiliations:** 1 Key Research Base of Humanities and Social Sciences of the Ministry of Education, Academy of Psychology and Behavior, Tianjin Normal University, Tianjin 300387, China; 2 Faculty of Psychology, Tianjin Normal University, Tianjin 300387, China; 3 Tianjin Key Laboratory of Student Mental Health and Intelligence Assessment, Tianjin 300387, China

**Keywords:** moral elevation, prosocial, children, implicit association test

## Abstract

This study investigated how moral elevation affects children’s prosocial behavior through two experiments. In Experiment 1 (*n* = 99; *M*_age_ = 10.48 ± 0.86 years), children were randomly assigned to Moral Elevation, Joy, or Neutral groups. Psychophysiological measures were recorded during both the baseline and task phases, while self-reported prosocial behavior was assessed using validated scales. Participants in the Moral Elevation group experienced emotional states marked by inspired, moved, touched, admiration, and uplifted, accompanied by a distinct pattern of sympathetic–parasympathetic coactivation. Although not statistically significant in self-reported measures, a notable pattern emerged wherein Moral Elevation yielded the highest prosocial scores, followed by Joy and then Neutral conditions. Experiment 2 (*n* = 92; *M*_age_ = 10.84 ± 0.76 years) employed a single-category Implicit Association Test (IAT) to assess prosocial behavioral tendencies. The Moral Elevation group exhibited a significantly stronger implicit prosocial bias on the IAT compared to both Joy and Neutral groups. These findings suggest that moral elevation possesses a unique emotional profile separate from general positive affect that activates dual dissociable pathways for children’s prosocial behavior: explicit and implicit processes. The study provides empirical support for incorporating moral elevation interventions in educational settings to cultivate integrated prosocial development.

## 1. Introduction

### 1.1. Moral Elevation

Moral elevation is a positive affective state elicited by witnessing others’ virtuous acts, formally defined as “an emotional response to perceived moral excellence” (Haidt, 2000, 2003). This state fundamentally arises from three interconnected components: moral beauty recognition, self-transcendent cognitive transformation, and heightened prosocial motivation (Haidt, 2003). It manifests as a composite of admiration, emotional resonance, and inspiration that collectively promote ethical emulation through other-oriented motivation rather than self-interest. The multidimensional structure of moral elevation integrates four domains. Affectively, its core phenomenology is characterized by an uplifted experience accompanied by profound feeling of tenderness and compassion (Freeman et al., 2009); Physiologically, it evokes somatic responses including cardiovascular arousal, thoracic warmth, and laryngeal constriction (Algoe & Haidt, 2009; Piper et al., 2015); Cognitively, it enhances beliefs in the inherent goodness of human nature and facilitates identification with moral exemplars (Algoe & Haidt, 2009; Bajović & Rizzo, 2020; Haidt, 2001; Krettenauer et al., 2013); Behaviorally, it motivates prosocial actions such as helping behaviors and charitable giving (Cox, 2010; Schnall et al., 2010).

Empirical evidence distinguishes moral elevation from general positive affect through its broader scope and enduring influence on prosociality (Algoe & Haidt, 2009; Cameron & Fredrickson, 2015). Longitudinal research indicates a 37% higher volunteer engagement rate three months post-induction compared to Neutral controls, whereas basic positive emotion inductions show no sustained impact (Cox, 2010). This sustainability advantage demonstrates moral elevation’s distinctive ability to convert transient affective states into enduring prosocial actions. Moreover, beyond its prosocial consequences, moral elevation contributes to ethical development and psychological well-being (Diessner et al., 2018; McGuire et al., 2020; Monroe, 2020).

Although research on moral elevation has predominantly focused on adults, recent studies have begun to examine how children experience this emotion. For instance, Gibhardt et al. (2024) used video-based stimuli and demonstrated that 6.5–8.5-year-olds’ moral elevation was elicited by observing prosocial behaviors. Additionally, Yarritu et al. (2025) used a video designed to induce moral elevation, and found that 10–15-year-olds’ social connectedness elicited by the video increased and that the experience triggered optimistic thoughts about humanity. These studies underscore that moral elevation is not only an emotionally uplifting experience but also a potential catalyst for fostering prosocial behavior in children. Therefore, it is necessary to further explore how children experience and respond to moral elevation, particularly the mechanisms linking this unique emotional state to their subsequent prosocial actions.

### 1.2. Autonomic Foundations of Moral Elevation

Psychophysiological research has begun to characterize the autonomic nervous system (ANS) underpinnings of moral elevation. Piper et al. (2015) identified the coactivation of the sympathetic and parasympathetic nervous systems as its distinctive signature. According to polyvagal theory (Porges, 1995, 2007), this unique physiological profile supports emotion regulation and facilitates affiliative behaviors, offering novel explanatory pathways for understanding moral elevation’s influence on individual actions.

Childhood represents a critical period for the development and consolidation of autonomic regulatory patterns (Porges & Furman, 2011). The parasympathetic system, indexed by measures such as respiratory sinus arrhythmia (RSA) and high-frequency heart rate variability (HF-HRV), undergoes progressive maturation during this time, evidenced by age-related increases in vagal tone (El-Sheikh et al., 2009; Beauchaine, 2015). This maturation fundamentally enhances children’s capacity for emotion regulation and adaptive social functioning (De Witte et al., 2016; Pergantis et al., 2025). Crucially, longitudinal evidence demonstrates a specific link between parasympathetic function and prosocial behaviors, highlighting age-dependent autonomic contributions to social engagement (Miller et al., 2017; Mastromatteo et al., 2024).

While studies in adults have established clear psychophysiological ANS responses specific to moral elevation (Piper et al., 2015), comparable evidence directly examining these ANS responses to moral elevation in school-aged children remains unconfirmed. Therefore, characterizing this response profile in children is a vital step for building a comprehensive neurophysiological model of moral emotion and for informing the design of targeted, neuroplasticity-based educational interventions.

Accordingly, the present study measured heart rate (HR), HF-HRV, and skin conductance level (SCL) as key indicators of autonomic coactivation during moral elevation in a sample of 9- to 12-year-old school-aged children. These measures are well-validated biomarkers of emotional reactivity in this pediatric age group (Pergantis et al., 2025).

### 1.3. Developmental Perspectives on Moral Elevation and Prosociality

Direct investigations into the impact of children’s moral elevation specifically on prosocial behavior remain limited. However, foundational studies reveal that even young children (ages 2–4) exhibit physiological and behavioral sensitivity to prosocial contexts. Using precise physiological measures, (Hepach et al., 2012) and (Hepach & Tomasello, 2020) demonstrated that toddlers (ages 2–4) display altruistic concern (indexed by pupil dilation) and positive affect (measured via upper-body posture) when observing others being helped. Building on this early-emerging sensitivity to prosocial contexts, late childhood (ages 7–11) develops advanced social learning capacities that support moral and prosocial development.

Moral development during these years is closely tied to cognitive and emotional maturation. Through peer interactions, observational learning, and reflective judgment, children progressively internalize moral standards and develop a more nuanced understanding of social norms (Banerjee, 2002; Bajović & Rizzo, 2020; Chai et al., 2024). According to Piaget’s cognitive developmental theory (Piaget, 1932, 1964), the emergence of reversible thinking allows children to analyze moral situations with greater flexibility and to view social rules as constructed rather than absolute. Concurrently, maturation of the prefrontal cortex supports self-regulation, inhibitory control, and emotion regulation—capacities essential for moral decision making and prosocial behavior (Diamond, 2013; Smetana, 2006).

Prosocial behavior operates through dual pathways: explicit (conscious) and implicit (automatic) processes (Aydinli et al., 2014). This distinction is particularly salient in late childhood, as children transition from heteronomous to autonomous moral reasoning (Cvencek et al., 2011; Dunham et al., 2008). Their prosocial tendencies often operate implicitly in this period, influenced by moral emotions and early socialization (Jiang et al., 2008; Lai et al., 2014; Vecchio et al., 2023). It is essential to assess both explicit and implicit measures to fully capture the influence of moral emotions. Tools such as the Implicit Association Test (IAT) are particularly useful for tapping into automatic associations that may not surface through self-report (Greenwald et al., 1998).

Therefore, to account for the dual pathways of prosociality and to account for developmental characteristics in children aged 9–12 years, the present research employed a multimethod approach across two experiments: one examining explicit prosocial behavior via self-report and the other probing implicit prosocial tendencies using the IAT.

### 1.4. The Present Study

Despite the well-documented effects of moral elevation on prosociality in adults, its physiological and behavioral manifestations in school-aged children remain understudied. Although recent developmental studies confirm that children can experience moral elevation (Gibhardt et al., 2024; Yarritu et al., 2025), no research has systematically investigated its characteristic ANS correlates in children aged 9–12 years. Furthermore, although moral elevation is linked to prosocial behavior (Haidt, 2003), evidence for its distinct effects on explicit versus implicit processes in children is scarce. It is crucial to examine its effects on both explicit and implicit levels. Therefore, to address these gaps, the present research utilized a psychophysiological approach to examine whether moral elevation, compared to both joy and neutral states, elicits distinct ANS responses and facilitates prosociality at both explicit and implicit levels in children across two experiments.

Based on the established theoretical and empirical foundations, the following hypotheses were tested:

(1) Moral elevation stimuli would elicit a pattern of autonomic coactivation, reflected in increased sympathetic nervous system activity (as measured by SCL) and increased parasympathetic influence (as indicated by HF-HRV), significantly differing from responses in both Joy and Neutral conditions.
(2) Moral elevation would lead to higher levels of explicitly self-reported prosocial outcomes compared to Joy and Neutral conditions.
(3) Children in the Moral Elevation condition would display a stronger implicit prosocial bias on the Implicit Association Test than those in the Joy or Neutral conditions.

## 2. Experiment 1

### 2.1. Methods

#### 2.1.1. Participants

Before data collection, a power analysis was conducted using G*Power 3.1 to determine the appropriate sample size. Assuming a medium effect size (*f* = 0.3), a significance level of α = 0.05, and a desired power of 0.80, the analysis indicated that a minimum of 90 participants would be required for sufficient statistical power.

Experiment 1 initially recruited 103 children aged 9–12 years (*M*_age_ = 10.48, *SD* = 0.86) from a local primary school. Through standardized quality control protocols, four participants were excluded due to missing key data (*n* = 1), comprehension check failures (*n* = 2), or excessive artifacts in physiological recordings (>30% signal loss; *n* = 1), resulting in a final sample of 99 participants (96.1% retention rate). These eligible participants were then systematically allocated to three experimental conditions: Moral Elevation (*n* = 31, 19 female), Joy (*n* = 34, 17 female), and Neutral (*n* = 34, 19 female). All participants met the inclusion criteria of right-handedness and absence of cardiovascular history. This study was approved by the ethics committee of the investigator’s institution.

#### 2.1.2. Materials

(1) Emotional self-assessment

Moral elevation was operationalized through a lexical decision task identifying core emotional lexicons (“Inspired”, “Moved”, “Touched”, “Admiration”, and “Uplifted”) with valence ratings quantified via a 7-point Likert scale (1 = not at all; 7 = extremely strong). Internal consistency was assessed using Cronbach’s α (Experiment 1: α= 0.844 at baseline, α = 0.798 during reactivity; Experiment 2: α = 0.881 at baseline, α = 0.829 during reactivity). A composite score was calculated by averaging these items. All Likert scale items were presented visually on paper forms using numerical scales. Before the assessment, the scoring rules were explained to the children, ensuring they understood the meaning of each emotion term and how to rate their feelings.

Basic emotions were assessed through a six-dimensional psychometric scale (“Peace”, “Joy”,” Fear”,” Anger”, “Sadness”, and “Disgust”) with valence ratings quantified via a 7-point Likert scale (1 = not at all; 7 = extremely strong), employing identical measurement procedures. Additionally, we counterbalanced the order of emotional items to avoid any potential order effects.

(2) Children’s prosocial behavior

This experiment adapted a self-report questionnaire assessing prosocial behavioral intentions in primary school students, integrating components from Liu’s (2012) *Prosocial Value Survey* and Yang’s (2012) *Children’s Prosocial Behavior Self-Evaluation Scale*. The instrument comprises four scenario-based items, each representing one of the core prosocial domains (Cooperate, Share, Help, and Comfort). All participants received identical items in fixed-order textual vignettes. Participants responded on a 0–10 scale (0 = no assistance, 10 = maximum possible effort), and total scores were calculated as the sum of all domain scores. To illustrate, the Help domain item presents a physical assistance scenario (e.g., After class, you see a classmate in the hallway struggling to carry 10 thick books, walking slowly toward the classroom. Seeing this, how many books would you assist in carrying?). Other domains target distinct prosocial dimensions: the Share domain involves allocating personally valued resources, the Cooperate domain centers on joint efforts toward group goals, and the Comfort domain addresses emotional support provision. This efficient design combines contextual specificity with quantifiable behavioral intentions to capture multidimensional prosociality.

(3) Emotionally induced videos

A multimodal video induction paradigm was implemented across three experimental conditions. The Moral Elevation group viewed a 270 s excerpt from *Touching China*, which shows an elderly man’s dedication to funding 300 underprivileged students through manual labor. The Joy condition utilized a 197 s comedic sequence from *Beware of Hot Tofu*, featuring humorous classroom and daily life scenarios involving a primary school student. Neutral controls observed a 183 s natural landscape video. All stimuli adhered to laboratory standards for emotional induction. The moral elevation video included a 90 s contextual introduction preceding the 180 s core induction sequence. All groups experienced equivalent active emotional stimulation periods of approximately 180 s. Following the *Chinese Emotional Video System* protocol (Xu et al., 2010), subjective emotional assessments confirmed successful elicitation of target affective states across conditions.

#### 2.1.3. Design and Procedure

Experiment 1 employed a 3 (group: Moral Elevation vs. Joy vs. Neutral) × 2 (phase: baseline vs. reactivity) mixed-design ANOVA. Child participants were voluntarily recruited and trained to use a 7-point Likert scale for emotional self-reporting.

**Figure 1:**
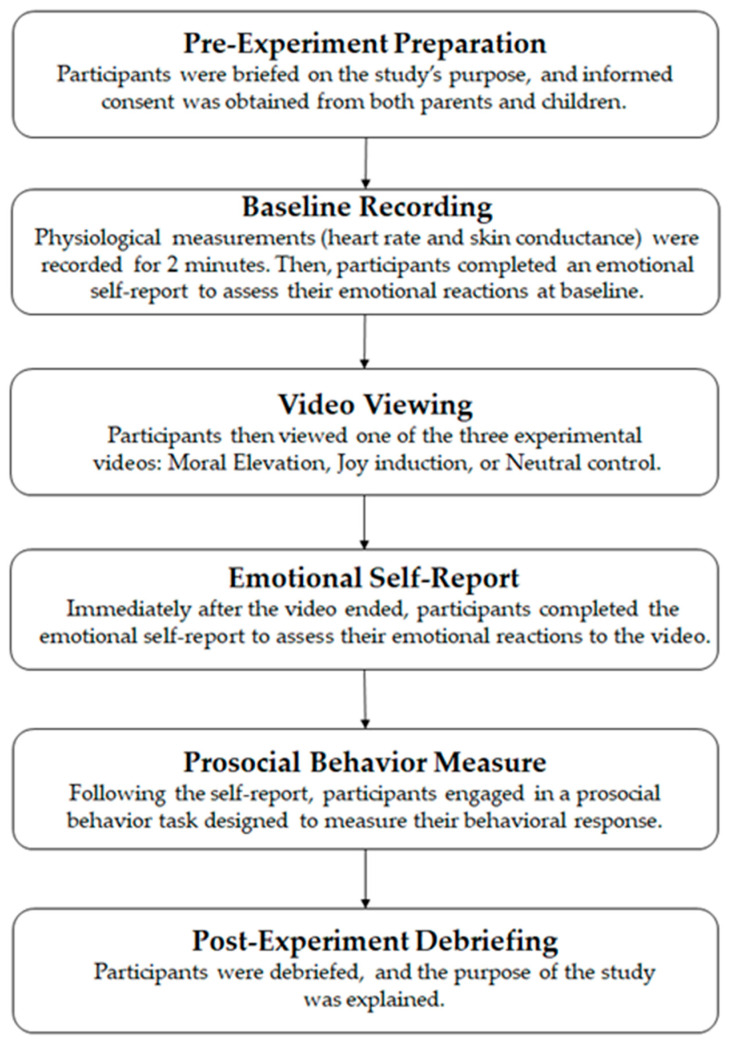
The procedure for Experiment 1.

The procedure for Experiment 1 is shown in Figure 1.

#### 2.1.4. Physiological Measures

During the experimental protocol, ANS activity was continuously monitored utilizing an MP160 16-channel physiological signal acquisition platform (BIOPAC Systems Inc.), implementing a sampling frequency of 2000 Hz. Electrocardiographic signals were acquired through the RSPEC-R transducer module, while electrodermal activity measurements were obtained via the PPGED-R sensor module. Primary data processing was conducted within the AcqKnowledge 5.0 environment, incorporating sequential procedures of baseline adjustment, signal denoising, and temporal segmentation. Subsequent quantitative analysis yielded three principal psychophysiological indices: tonic Skin Conductance Level (SCL), mean Heart Rate (HR), and High-Frequency component of Heart Rate Variability (HF-HRV). To ensure compliance with parametric test requirements, HF-HRV metrics underwent natural logarithmic transformation before statistical evaluation.

### 2.2. Results

We examined participants’ subjective emotional experiences and autonomic arousal responses before and after viewing different video materials. The means and standard deviations for the target emotional dimensions are shown in Table 1.

#### 2.2.1. Differences in Subjective Emotional Experiences

To examine the emotional induction effects of the videos in Experiment 1, a series of 3 (group: Moral Elevation, Joy, Neutral) × 2 (phase: baseline vs. reactivity) repeated-measures ANOVAs was conducted for basic emotional categories (Peace, Joy, Fear, Anger, Sadness, and Disgust). The results in Table 1 revealed the following.

For Peace, both the group effect, *F*(2, 96) = 4.55, *p* = 0.013, $\eta_{p}^{2}$ = 0.087, and the phase effect, *F*(1, 96) = 30.53, *p* < 0.001, $\eta_{p}^{2}$ = 0.241, were significant, along with a significant interaction, *F*(2, 96) = 10.53, *p* < 0.001, $\eta_{p}^{2}$ = 0.180. For Joy, the group effect, *F*(2, 96) = 10.95, *p* < 0.001, $\eta_{p}^{2}$ = 0.186, and interaction, *F*(2, 96) = 29.62, *p* < 0.001, $\eta_{p}^{2}$ = 0.382, were significant. For Sadness, all effects were significant: group, *F*(2, 96) = 12.29, *p* < 0.001, $\eta_{p}^{2}$ = 0.204; phase, *F*(1, 96) = 17.28, *p* < 0.001, $\eta_{p}^{2}$ = 0.153; and interaction, *F*(2, 96) = 11.15, *p* < 0.001, $\eta_{p}^{2}$ = 0.189.

Simple effects analyses indicated that at baseline, there were no significant group differences in Peace, Joy, or Sadness. During the reactivity phase, the Moral Elevation and Joy groups both exhibited significant decreases in Peace. The Joy group also showed a significant increase in Joy, while the Moral Elevation group reported increased Sadness. The Neutral group displayed no significant emotional changes across phases.

For the remaining emotions, Fear showed a significant group effect only, *F*(2, 96) = 3.61, *p* = 0.031, $\eta_{p}^{2}$ = 0.070. Disgust showed a significant phase effect, *F*(1, 96) = 4.45, *p* = 0.037, $\eta_{p}^{2}$ = 0.044. Anger showed no significant effects.

For Moral Elevation, a paired-sample *t*-test was conducted to compare Moral Elevation between the baseline and reactivity phases within the Moral Elevation group. Results showed that participants reported significantly higher levels of moral elevation during the reactivity phase compared to baseline, *t*(30) = −9.01, *p* < 0.001, Cohen’s *d* = −1.618.

These results confirm that the selected video materials effectively induced distinct emotional states across conditions and validate the success of the emotional manipulation in Experiment 1.

#### 2.2.2. Differences in Physiological Arousal Indicators

To examine the effects of moral elevation on ANS activity, a series of 3 (group: Moral Elevation, Joy, Neutral) × 2 (phase: baseline vs. reactivity) repeated-measures ANOVAs was conducted for each physiological indicator: SCL, HR, and HF-HRV. Results in Table 1 revealed the following.

For SCL, the main effect of phase was significant, *F*(1, 96) = 38.88, *p* < 0.001, $\eta_{p}^{2}$ = 0.288, while group and interaction effects were not significant. Post hoc comparisons revealed that both the Moral Elevation group, *t*(98) = −4.70, *p* < 0.001, Cohen’s *d* = −0.283, and the Joy group, *t*(98) = −4.09, *p* = 0.001, Cohen’s *d* = −0.235, exhibited significantly increased SCL during the reactivity phase, whereas the Neutral group showed no change (*p* = 0.684).

For HR, a significant main effect of phase was observed, *F*(1, 96) = 9.67, *p* = 0.002, $\eta_{p}^{2}$ = 0.092, along with a significant group × phase interaction, *F*(2, 96) = 3.27, *p* = 0.042, $\eta_{p}^{2}$ = 0.064. To explore this interaction, simple effects analyses were conducted for each group. In the Moral Elevation group, HR increased significantly from baseline to the reactivity phase, *F*(1, 96) = 15.97, *p* < 0.001. No significant changes in HR were observed in the Joy group *(p* = 0.111) or the Neutral group (*p* = 0.978).

For HF-HRV, neither group nor phase main effects were significant, but the group × phase interaction was, *F*(2, 96) = 3.49, *p* = 0.035, $\eta_{p}^{2}$ = 0.068, indicating differential changes in HF-HRV across groups from baseline to the reactivity phase. To explore this interaction, simple effects analyses were conducted for each group. In the Moral Elevation group, HF-HRV increased significantly from baseline to the reactivity phase, *F*(1, 96) = 8.12, *p* = 0.008. No significant changes in HF-HRV were observed in the Joy group (*p* = 0.409) or the Neutral group (*p* = 0.135).

Together, these results suggest that moral elevation uniquely elicited a pattern of sympathetic (SCL, HR) and parasympathetic (HF-HRV) coactivation, distinguishing it from Joy and Neutral conditions, which lacked such dual modulation.

#### 2.2.3. Differences in Prosocial Behavior Scores

**Figure 2:**
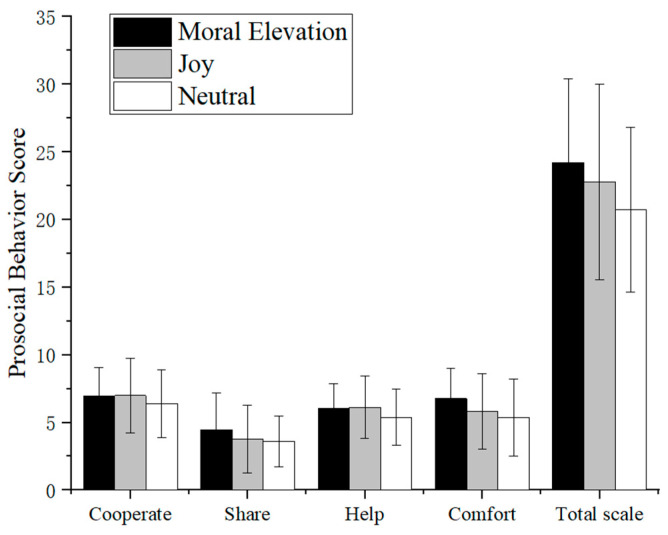
Prosocial behavior scores in Moral Elevation, Joy, and Neutral Groups.

Figure 2 presents the prosocial behavior scores across the Moral Elevation, Joy, and Neutral groups for each subscale (Cooperate, Share, Help, Comfort) and the total prosocial behavior score. The error bars represent the standard deviations of the scores across the groups.

A one-way ANOVA was conducted with group as the independent variable and four prosocial behavior subtypes plus total prosocial score as dependent variables. Results revealed that the Moral Elevation group demonstrated the highest scores in Share, Comfort, and total prosocial behavior, with the Joy group scoring higher than the Neutral group. However, no statistically significant intergroup differences were observed across emotional conditions. *F*_Cooperate_ (2, 96) = 0.56, *p* = 0.572; *F*_Share_ (2, 96) = 1.17, *p* = 0.316; *F*_Help_ (2, 96) = 1.31, *p* = 0.274; *F*_Comfort_ (2, 96) = 2.38, *p* = 0.098; *F*_Total_ (2, 96) = 2.37, *p* = 0.099.

### 2.3. Discussion

Experiment 1 provided the first empirical demonstration that moral elevation elicited a distinct autonomic coactivation pattern, characterized by simultaneous sympathetic and parasympathetic activation, in school-aged children. This response, reflected by significant increases in both SCL and HF-HRV, closely mirrored the psychophysiological signature previously documented in adults (Piper et al., 2015). The identification of this pattern in a child sample represents a novel contribution to the developmental literature, confirming that the neurophysiological architecture underlying moral elevation is already operational by late childhood. While general positive arousal (as induced by joy) also elevated sympathetic markers (SCL and HR), only moral elevation produced the concomitant increase in parasympathetic influence (HF-HRV), supporting its theoretical distinction from nonspecific positive affect. This replication of adult-like coactivation in a younger population significantly advances our understanding of the neurophysiological continuity of moral emotions across development.

The observed convergence of increased parasympathetic and sympathetic activity during moral elevation may create an optimal physiological context for prosociality in children. Parasympathetic activation supports the social–emotional understanding and regulatory capacity necessary for perceiving others’ needs and responding appropriately. Meanwhile, sympathetic activation provides the motivational energy and readiness to enact helping behaviors (Porges, 2007; Mastromatteo et al., 2024). This integrated autonomic state, which accompanies the emotional experience of moral elevation, may enhance children’s readiness and ability to translate moral feelings into concrete prosocial actions.

Although the self-reported prosocial scores did not reach statistical significance across groups, a consistent pattern emerged wherein the Moral Elevation group showed the highest prosocial scores, followed by the Joy and then the Neutral conditions. The absence of significant group differences in explicit prosocial self-reports may reflect inherent limitations of the self-report instrument for this age group, rather than the absence of prosocial change. On the one hand, children’s self-reports are vulnerable to experimenter expectations, such as “good kid” responses (Schwartz & Howard, 1984). Moreover, social desirability bias, where children aim to provide morally acceptable responses regardless of experimental condition (Banerjee, 2002), potentially obscures group-level differences and results in uniformly high scores across conditions. On the other hand, the developmental stage of the participants (aged 9–12) is a critical factor contributing to the instability of explicit measures. Although adapted for child comprehension, scenario-based self-ratings require children to integrate internal emotional states with abstract hypothetical reasoning, which may exceed the optimal cognitive load for children aged 9–12. The development of children’s social emotionalization depends on the maturity of the prefrontal lobe (Eslinger et al., 2021), which is still undergoing maturation at this age (Sowell et al., 2004). This prefrontal cortex immaturity may further contribute to the instability of explicit prosocial decisions. Additionally, explicit self-reports are often influenced by meta-cognitive abilities (Carlo et al., 2003). As a result, self-reports of prosocial behavior may not fully capture underlying automatic or unconscious prosocial tendencies, particularly following emotional inductions.

Consequently, given the limitations of explicit self-reports and the developmental context, implicit prosocial motivation is likely a more stable indicator and may better reflect the underlying prosocial behavior tendency of children affected by moral elevation (Aydinli et al., 2014). Therefore, in the subsequent study (Experiment 2), we employed the IAT (Greenwald et al., 1998) to measure implicit prosocial associations. The IAT avoids the issues plaguing self-reports by measuring reaction-time differences in compatible vs. incompatible tasks (Cai, 2003), providing more reliable insight into implicit social cognition related to prosociality (Christner et al., 2022). This approach offers a crucial perspective on the potential influence of moral elevation in children, laying the groundwork for examining its effects on implicit prosocial motivation.

In summary, Experiment 1 identified a sympathetic-parasympathetic coactivation pattern specific to moral elevation in children. Although its translation to explicit prosociality was constrained by neurodevelopmental variability and measurement limitations. These findings underscore the need for integrative frameworks that concurrently assess both explicit behaviors and implicit processes in moral emotion research.

## 3. Experiment 2

### 3.1. Methods

#### 3.1.1. Participants

Before data collection, a power analysis was conducted using G*Power 3.1 to determine the appropriate sample size. Assuming a medium effect size (*f* = 0.3), a significance level of α = 0.05, and a desired power of 0.80, the analysis indicated that a minimum of 90 participants would be required for sufficient statistical power.

Experiment 2 recruited 100 children aged 9–12 years (*M*_age_ = 10.84, *SD* = 0.76) from a local primary school. After implementing standardized quality control, eight participants were excluded due to missing key data (*n* = 2), comprehension check failures (*n* = 3), IAT error rates >20% (*n* = 2), or >10% of reaction times exceeding 300–3000 ms thresholds (*n* = 1), yielding a final sample of 92 participants (92% retention rate). These right-handed participants without cardiovascular history were systematically allocated to three conditions: Moral Elevation (*n* = 31, 19 female), Joy (*n* = 32, 13 female), and Neutral (*n* = 29, 18 female). All participants met the inclusion criteria of right-handedness and absence of cardiovascular history. This study was approved by the ethics committee of the investigator’s institution.

#### 3.1.2. Materials

The materials of emotional self-assessment and emotionally induced videos are the same as Experiment 1.

#### 3.1.3. Design and Procedure

Experiment 2 employed a 3 (group: Moral Elevation vs. Joy vs. Neutral) × 2 (phase: baseline vs. reactivity) mixed factorial design. Participants were voluntarily recruited children and trained to use a 7-point Likert scale for emotional self-reporting.

The procedure comprised two sequential phases. First, during the resting phase, participants sat still for two minutes while baseline measures were recorded, followed by completion of self-report measures. Second, in the task phase, participants were randomly assigned to view one of three video stimuli: Moral Elevation (*n* = 31), Joy (*n* = 32), or Neutral (*n* = 29). After viewing, they completed post-exposure assessments. The protocol concluded with an IAT measuring prosocial behavioral tendencies (He & Zhu, 2016). All experimental conditions maintained standardized administration protocols for self-report data collection.

The IAT in our study assessed automatic associations toward prosocial behavior using a child-adapted paradigm. Participants categorized target words into conceptual–attribute pairs by pressing designated keys. The task contrasted compatible trials (e.g., Self–Altruism or Other–Nonaltruism) with incompatible trials (e.g., Self–Nonaltruism or Other–Altruism), operationalizing cognitive consistency versus conflict through differences in reaction times. The specific procedural details and stimulus materials are outlined in Appendix A.

To ensure comprehension among children aged 9–12, a multistep protocol was applied. Instructions were presented both verbally and visually in age-appropriate language, followed by practice trials with real-time feedback. Participants were required to verbally explain the rules and achieve at least 80% accuracy in practice before proceeding to the formal task. Those who did not meet the criteria repeated the instruction–practice cycle until successful.

### 3.2. Results

#### 3.2.1. Subjective Emotional Experiences

We examined participants’ subjective emotional experiences before and after viewing different video materials. The means and standard deviations for the target emotional dimensions are shown in Table 2.

To examine the emotional induction effects of the videos in Experiment 2, a series of 3 (group: Moral Elevation, Joy, Neutral) × 2 (phase: baseline vs. reactivity) repeated-measures ANOVAs were conducted for basic emotional category (Peace, Joy, Fear, Anger, Sadness, and Disgust), and the results in Table 2 revealed the following.

For Peace, the phase effect, *F*(1, 89) = 35.17, *p* < 0.001, $\eta_{p}^{2}$ = 0.283, and interaction, *F*(2, 89) = 9.604, *p* < 0.001, $\eta_{p}^{2}$ = 0.178, were significant. For Joy, the group effect, *F*(2, 89) = 8.86, *p* < 0.001, $\eta_{p}^{2}$ = 0.166, and interaction, *F*(2, 89) = 24.12, *p* < 0.001, $\eta_{p}^{2}$ = 0.352, were significant. For Sadness, the group effect, *F*(2, 89) = 4.278, *p* = 0.017, $\eta_{p}^{2}$ = 0.088, and phase, *F*(1, 89) = 8.764, *p* = 0.004, $\eta_{p}^{2}$ = 0.089, were significant.

Simple effects and post hoc analyses indicated that at baseline, there were no significant group differences in Peace, Joy, or Sadness. During the reactivity phase, the Moral Elevation and Joy groups both exhibited significant decreases in Peace. The Joy group also showed a significant increase in joy, while the Moral Elevation group reported increased Sadness. The Neutral group displayed no significant emotional changes across phases.

For the remaining emotions, Fear, Anger, and Disgust showed no significant effects.

For moral elevation, a paired-sample *t*-test was conducted to compare moral elevation between the baseline and reactivity phases within the Moral Elevation group. Results showed that participants reported significantly higher levels of moral elevation during the reactivity phase compared to baseline, *t*(30) = −8.67, *p* < 0.001, Cohen’s *d* = −1.558.

These results confirm that the selected video materials effectively induced distinct emotional states across conditions and validate the success of the emotional manipulation in Experiment 2.

#### 3.2.2. Prosocial Behavior

RTs were truncated to 300–3000 ms boundaries and log-transformed to normalize the distribution. Congruent trials (pairing target–attribute concepts consistent with implicit attitudes) and incongruent trials (inconsistent pairings) were operationally defined. The IAT effect was calculated by subtracting the mean RTs of congruent trials from incongruent trials. Complete statistical outcomes are presented in Table 3.

The paired-sample *t*-test revealed a significant reaction time difference between compatible and incompatible tasks, *t*(91) = −11.03, *p* < 0.001, Cohen’s *d* = −1.15, indicating faster responses in compatible tasks and confirming the IAT effect. A one-way ANOVA demonstrated a significant main effect of group on IAT effect values, *F*(2,89) = 4.04, *p* = 0.021, $\eta_{p}^{2}$ = 0.083. Post hoc comparisons showed significantly greater IAT effects in the Moral Elevation group compared to Joy, *t*(91) = 2.55, *p* = 0.033, Cohen’s *d* = 0.642, and trending toward significance compared to Neutral group, *t*(91) = 2.36, *p* = 0.053, Cohen’s *d* = 0.609. There are no significant differences observed between the Joy and Neutral groups. No other significant differences were detected.

### 3.3. Discussion

Experiment 2 applied the IAT to assess implicit prosocial tendencies. This approach minimizes social desirability bias and explicit reporting limitations common in child samples (Cai, 2003). Subjective emotion reports confirmed that video materials successfully elicited the target emotions. The Moral Elevation group showed significantly faster responses during compatible trials (pairing prosocial concepts with self) versus incompatible trials. This reaction time difference confirms automatic activation of implicit prosociality (Frith & Frith, 2008), with the significantly greater IAT effect in the Moral Elevation group relative to both Joy and Neutral conditions indicating that Moral Elevation uniquely drives implicit prosociality beyond general positive affect.

The design of IAT paired self-referential categories (e.g., “I”, “myself”) with prosocial attributes (e.g., “help”, “share”) to assess automatic associations between self-identity and prosociality. This approach is developmentally appropriate for school-aged children, who are increasingly internalizing moral standards and refining their self-concept through growing self-reflection and social awareness (Piaget, 1964; Krettenauer et al., 2013). As moral values become integrated into their self-schema, children form a more coherent moral self-identity (Christner et al., 2022). The IAT captures this link by measuring the strength of automatic associations between the self and prosocial concepts. Stronger self-prosocial associations reflect a developmental shift from externally guided to more autonomous, self-integrated moral motivation (Aydinli et al., 2014; Tangney et al., 2007), illustrating how moral emotions such as elevation may promote implicit prosocial behavior through self-referential pathways.

The enhanced implicit prosocial bias observed on the IAT reflects the activation of self-integrated moral pathways. This finding aligns with Moral Emotions Theory, which links moral affective states to distinct prosocial motivations (Tangney et al., 2007). Crucially, the sympathetic-parasympathetic coactivation observed during moral elevation in Experiment 1 provides a physiological basis for this effect. This specific autonomic pattern likely facilitates the internalization of moral affect into the self-concept, thereby strengthening the automatic association between the self and prosociality measured by the IAT.

In summary, Experiment 2 confirmed moral elevation’s specific role in promoting children’s implicit prosociality. These findings reveal that educators can foster children’s prosocial development through implicit pathways by aligning moral emotions with self-concept exploration. Such integration may bridge implicit tendencies to explicit actions.

## 4. General Discussion

This study employed a multimodal approach integrating psychophysiological and behavioral paradigms to investigate moral elevation in primary school students. The main aims were to examine subjective emotional responses and ANS dynamics during moral elevation, while assessing its distinct behavioral pathways through parallel measurement of explicit (Experiment 1) and implicit (Experiment 2) prosocial responses relative to general positive affect.

Our findings provided the first evidence that moral elevation simultaneously activates both sympathetic and parasympathetic nervous system activity in 9-to-12-year-old children, manifesting as a coactivation pattern characterized by marked increases in HF-HRV and SCL. Hence, the present results demonstrated high consistency with autonomic response patterns observed in former studies (Piper et al., 2015; Mastromatteo et al., 2024). This correspondence indicates that children already possess neurophysiological foundations for moral emotion processing similar to adults.

This coactivation pattern was specifically associated with moral elevation and not observed in response to general positive affect (joy), providing a novel neurophysiological basis for differentiating moral elevation from nonspecific positive emotions. From the perspective of Polyvagal Theory (Porges, 2007), this neurophysiological profile potentially facilitates prosocial behavior through two complementary mechanisms: sympathetic-driven emotional responsiveness to moral stimuli and parasympathetic-mediated cognitive integration with reduced behavioral inhibition. Together, they establish a neurophysiological state supportive of social engagement, wherein ventral vagal activation heightens sensitivity to others’ needs, and sympathetic arousal mobilizes energy for prosocial behaviors (Porges, 2007). Thus, the specific autonomic pattern during moral elevation offers a mechanistic explanation for its superior efficacy in enhancing implicit prosocial tendencies compared to joy, as it effectively bridges moral affect with the neurophysiological readiness for other-oriented action.

Notably, this distinct physiological signature corresponded with a dissociation in behavioral outcomes. While neither the Moral Elevation group nor the Joy group showed significantly higher self-reported explicit prosocial behavior than the Neutral group (despite a nonsignificant trend of Moral Elevation > Joy > Neutral), moral elevation significantly enhanced children’s implicit prosocial tendencies, with elevated IAT effect scores surpassing both Joy and Neutral groups.

To further understand the dissociation between implicit and explicit prosocial responses, we consider the role of executive function (EF) and emotional competence development during late childhood. EF, which includes inhibition, cognitive flexibility, and working memory, undergoes significant development in children aged 9–12 (Diamond, 2013; Zelazo & Carlson, 2012). These cognitive functions critically support emotion regulation and social decision making, processes essential for translating moral emotions like moral elevation into prosocial actions. In our study, the observed behavioral dissociation may stem from the ongoing maturation of emotion regulation during this developmental stage. Additionally, emotional competence, defined as the ability to perceive, understand, and manage emotions (Denham, 2006), is fundamental to prosocial development (Yang, 2012). This explains why implicit prosocial tendencies remain stable in late childhood, while explicit expression of these tendencies may be limited by immature emotional and social decision-making systems. This complex interplay between EF maturation and emotional competence development explains the implicit–explicit discrepancy observed in our findings. This developmental progression resonates with Piaget’s conceptualization of the heteronomous-to-autonomous transition, wherein children gradually evolve from relying on external moral constraints to developing self-guided moral principles (Piaget, 1964). Thus, while moral emotions may activate early intrinsic motivation (reflecting autonomous inclination), the full behavioral enactment of prosociality depends on later-maturing integrative capacities such as hot EF and emotion regulation, which are still consolidating in late childhood.

While this study reveals the impact of moral elevation on children’s prosocial behavior and its ANS correlates through psychophysiological experiments, some limitations of the study must be considered. First, despite assessing multiple prosocial domains (collaboration, sharing, helping, and comforting) via self-reports, future work should implement ecologically valid behavioral paradigms (e.g., sticker-sharing tasks; Chai et al., 2024) to minimize recall bias and social desirability effects (Jiang et al., 2008). Second, since 9–12-year-olds experience ongoing development in moral reasoning and self-representation (Bajović & Rizzo, 2020; Krettenauer et al., 2013), longitudinal designs become essential to track how moral elevation’s motivational role emerges during cognitive maturation. Third, to ensure conceptual precision, moral elevation-specific emotions were assessed exclusively in the moral elevation condition. While preventing contamination from phenomenologically distinct states in the Joy/Neutral groups, this design limits direct cross-condition comparisons. Future studies should, therefore, implement systematic measurement across induction contexts to advance discriminant validity testing of moral emotion specificity. Finally, although this study identified distinctive ANS activation patterns during moral elevation, future neuroimaging research should map the underlying neurophysiological pathways to fully characterize the neural substrates regulating prosocial behavior.

## 5. Conclusions

This study systematically investigated the autonomic mechanisms and behavioral effects of moral elevation in 9–12-year-old children. We found that moral elevation simultaneously activated both the sympathetic nervous system (SCL) and the parasympathetic nervous system (HRV). This coactivation pattern mirrors adult responses, confirming children’s well-developed neurophysiological capacity for moral emotions. Behaviorally, moral elevation significantly enhanced implicit prosocial tendencies while revealing a dissociation between explicit and implicit prosocial behaviors. This demonstrated how moral elevation drives moral conduct through automated processing pathways. These findings provide crucial evidence for intrinsic moral education approaches that cultivate emotional resonance.

## Figures and Tables

**Table 1:** Subjective experience of emotions induced by video and autonomic nervous activity (*M* ± *SD*).

	Moral Elevation		Joy		Neutral	
	Baseline	Reactivity	Baseline	Reactivity	Baseline	Reactivity
Basic emotion						
Peace	**4.48 ± 1.36**	**3.00 ± 1.59**	**4.44 ± 1.62**	**3.09 ± 1.36**	4.47 ± 1.31	4.62 ± 1.60
Joy	**3.77 ± 2.05**	**1.71 ± 1.37**	**3.71 ± 1.78**	**5.00 ± 1.74**	3.58 ± 1.60	4.00 ± 1.39
Fear	1.23 ± 0.50	1.13 ± 0.50	1.09 ± 0.29	1.03 ± 0.17	1.56 ± 1.24	1.32 ± 0.88
Anger	1.19 ± 0.75	1.10 ± 0.30	1.15 ± 0.61	1.10 ± 0.29	1.12 ± 0.69	1.12 ± 0.54
Sadness	**1.32 ± 0.95**	**2.32 ± 1.62**	1.03 ± 0.17	1.03 ± 0.17	1.09 ± 0.38	1.24 ± 0.74
Disgust	1.07 ± 0.36	1.00 ± 0.00	1.15 ± 0.61	1.00 ± 0.00	1.18 ± 0.72	1.06 ± 0.24
Moral elevation	**1.72 ± 1.07**	**3.85 ± 1.52**				
Inspired	**1.97 ± 1.38**	**2.84 ± 1.90**				
Moved	**1.74 ± 1.39**	**4.61 ± 2.03**				
Touched	**1.94 ± 1.59**	**4.16 ± 2.07**				
Admiration	**1.52 ± 1.21**	**4.45 ± 2.28**				
Uplifted	**1.45 ± 1.23**	**3.19 ± 1.94**				
Physiological arousal						
SCL (µs)	**8.73 ± 6.28**	**10.28 ± 7.13**	**9.27 ± 4.55**	**10.56 ± 4.49**	**8.32 ± 4.89**	**8.94 ± 5.37**
HR (BPM)	**89.90 ± 9.68**	**92.37 ± 9.62**	88.88 ± 8.50	90.11 ± 8.48	86.79 ± 10.24	86.77 ± 10.44
HF-HRV	**3.82 ± 1.06**	**4.36 ± 1.17**	4.36 ± 1.30	4.08 ± 1.81	4.43 ± 1.21	4.27 ± 1.12

Notes: SCL = skin conductance level, HR = heart rate, HF-HRV = high-frequency heart rate variability. **Bold** values indicate significant baseline-to-reactivity changes (paired-sample *t*-tests, *p* < 0.05).

**Table 2:** Subjective experience of emotions induced by video (*M* ± *SD*).

	Moral Elevation		Joy		Neutral	
	Baseline	Reactivity	Baseline	Reactivity	Baseline	Reactivity
Basic emotion						
Peace	**4.65 ± 1.43**	**3.23 ± 1.54**	**5.03 ± 1.56**	**3.34 ± 1.58**	**4.45 ± 1.38**	**4.48 ± 1.57**
Joy	**4.13 ± 1.89**	**2.32 ± 1.58**	**4.25 ± 1.76**	**5.34 ± 1.60**	3.52 ± 1.81	3.97 ± 1.80
Fear	1.39 ± 0.92	1.36 ± 1.17	1.09 ± 0.30	1.00 ± 0.00	1.28 ± 0.65	1.28 ± 0.80
Anger	1.36 ± 0.88	1.23 ± 0.34	1.00 ± 0.00	1.00 ± 0.00	1.34 ± 0.74	1.10 ± 0.56
Sadness	**1.42 ± 0.99**	**1.87 ± 1.41**	1.09 ± 0.53	1.13 ± 0.55	1.10 ± 0.41	1.31 ± 0.81
Disgust	1.42 ± 1.21	1.26 ± 1.23	1.03 ± 0.18	1.00 ± 0.00	1.72 ± 0.76	1.07 ± 0.26
Moral elevation	**2.21 ± 1.30**	**4.35 ± 1.41**				
Inspired	**2.68 ± 1.56**	**3.65 ± 1.45**				
Moved	**1.90 ± 1.56**	**4.65 ± 1.98**				
Touched	**2.23 ± 1.61**	**4.68 ± 1.90**				
Admiration	**2.26 ± 1.69**	**4.84 ± 1.99**				
Uplifted	**1.97 ± 1.49**	**3.94 ± 1.79**				

Note: **Bold** values indicate significant baseline-to-reactivity changes (paired-sample *t*-tests, *p* < 0.05).

**Table 3:** Implicit prosocial behavior (*M* ± *SD*).

	Moral Elevation (*n* = 31)		Joy (*n* = 32)		Neutral (*n* = 29)	
	RT (ms)	log	RT (ms)	log	RT (ms)	log
**Compatible**	939 ± 274	6.81 ± 0.27	1007 ± 217	6.90 ± 0.20	996 ± 223	6.88 ± 0.20
**Incompatible**	1387 ± 272	7.22 ± 0.19	1287 ± 276	7.14 ± 0.21	1286 ± 291	7.14 ± 0.22
**IAT**	447 ± 286	0.41 ± 0.25	247 ± 326	0.25 ± 0.25	291 ± 302	0.25 ± 0.26

Notes: RT = reaction time in milliseconds; IAT = Implicit Association Test. IAT = RT_Incompatible_ − RT_Compatible_, logIAT = logRT_Incompatible_ − logRT_Compatible._ Higher logIAT values indicate stronger implicit prosocial associations.

## Data Availability

The original data presented in the study are openly available via the OSF at https://osf.io/rbx47/?view_only=4929fd6855584da581aeffdc3ee2111c (accessed on 30 June 2025).

## Appendix A

**Table A1:** Implicit Association Test steps and presentation materials.

Step	Task Description	(Left Upper Side) Label (Right Upper Side)	Presentation Materials
1	Attribute Word Discrimination	Self-Other	Self or Other Vocabulary
2	Initial Target Word Discrimination	Altruism-Nonaltruism	Altruism or Nonaltruism Vocabulary
3	Compatible Task (Practice)	Altruism+Self-Nonaltruism + Other	All Vocabulary
4	Compatible Task (Test)	Altruism+Self-Nonaltruism + Other	All Vocabulary
5	Reverse Target Word Discrimination	Nonaltruism-Altruism	Nonaltruism or Altruism Vocabulary
6	Incompatible Task (Practice)	Altruism+Other-Nonaltruism + Self	All Vocabulary
7	Incompatible Task (Test)	Altruism+Other-Nonaltruism + Self	All Vocabulary

## References

[B1-behavsci-15-01246] Algoe S. B., Haidt J. (2009). Witnessing excellence in action: The ‘other-praising’ emotions of elevation, gratitude, and admiration. The Journal of Positive Psychology.

[B2-behavsci-15-01246] Aydinli A., Bender M., Chasiotis A., Cemalcilar Z., Van de Vijver F. J. (2014). When does self-reported prosocial motivation predict helping? The moderating role of implicit prosocial motivation. Motivation and Emotion.

[B3-behavsci-15-01246] Bajović M., Rizzo K. (2020). Meta-moral cognition: Bridging the gap among adolescents’ moral thinking, moral emotions and moral actions. International Journal of Adolescence and Youth.

[B4-behavsci-15-01246] Banerjee R. (2002). Children’s understanding of self-presentational behavior: Links with mental-state reasoning and the attribution of embarrassment. Merrill-Palmer Quarterly.

[B5-behavsci-15-01246] Beauchaine T. P. (2015). Respiratory sinus arrhythmia: A transdiagnostic biomarker of emotion dysregulation and psychopathology. Current Opinion in Psychology.

[B6-behavsci-15-01246] Cai H. J. (2003). A review on implicit association test. Advances in Psychological Science.

[B7-behavsci-15-01246] Cameron C. D., Fredrickson B. L. (2015). Mindfulness facets predict helping behavior and distinct helping-related emotions. Mindfulness.

[B8-behavsci-15-01246] Carlo G., Hausmann A., Christiansen S., Randall B. A. (2003). Sociocognitive and behavioral correlates of a measure of prosocial tendencies for adolescents. Journal of Early Adolescence.

[B9-behavsci-15-01246] Chai Q., Yin J., Shen M., He J. (2024). Act generously when others do so: Majority influence on young children’s sharing behavior. Developmental Science.

[B10-behavsci-15-01246] Christner N., Pletti C., Paulus M. (2022). How does the moral self-concept relate to prosocial behaviour? Investigating the role of emotions and consistency preference. Cognition and Emotion.

[B11-behavsci-15-01246] Cox K. S. (2010). Elevation predicts domain-specific volunteerism 3 months later. The Journal of Positive Psychology.

[B12-behavsci-15-01246] Cvencek D., Greenwald A. G., Meltzoff A. N. (2011). Measuring implicit attitudes of 4-year-olds: The preschool implicit association test. Journal of Experimental Child Psychology.

[B13-behavsci-15-01246] Denham S. A. (2006). Social-emotional competence as support for school readiness: What is it and how do we assess it?. Early Education and Development.

[B14-behavsci-15-01246] De Witte N. A. J., Sutterlin S., Braet C., Mueller S. C. (2016). Getting to the heart of emotion regulation in youth: The role of interoceptive sensitivity, heart rate variability, and parental psychopathology. PLoS ONE.

[B15-behavsci-15-01246] Diamond A. (2013). Executive functions. Annual Review of Psychology.

[B16-behavsci-15-01246] Diessner R., Pohling R., Stacy S., Güsewell A. (2018). Trait appreciation of beauty: A story of love, transcendence, and inquiry. Review of General Psychology.

[B17-behavsci-15-01246] Dunham Y., Baron A. S., Banaji M. R. (2008). The development of implicit intergroup cognition. Trends in Cognitive Sciences.

[B18-behavsci-15-01246] El-Sheikh M., Kouros C. D., Erath S., Cummings E. M., Keller P., Staton L. (2009). Marital conflict and children’s externalizing behavior: Interactions between parasympathetic and sympathetic nervous system activity. Monographs of the Society for Research in Child Development.

[B19-behavsci-15-01246] Eslinger P. J., Anders S., Ballarini T., Boutros S., Krach S., Mayer A. V., Moll J., Newton T. L., Schroeter M. L., de Oliveira-Souza R., Raber J., Sullivan G. B., Swain J. E., Lowe L., Zahn R. (2021). The neuroscience of social feelings: Mechanisms of adaptive social functioning. Neuroscience & Biobehavioral Reviews.

[B20-behavsci-15-01246] Freeman D., Aquino K., McFerran B. (2009). Overcoming beneficiary race as an impediment to charitable donations: Social dominance orientation, the experience of moral elevation, and donation behavior. Personality and Social Psychology Bulletin.

[B21-behavsci-15-01246] Frith C. D., Frith U. (2008). Implicit and explicit processes in social cognition. Neuron.

[B22-behavsci-15-01246] Gibhardt S., Hepach R., Henderson A. M. E. (2024). Observing prosociality and talent: The emotional characteristics and behavioral outcomes of elevation and admiration in 6.5- to 8.5-year-old children. Frontiers in Psychology.

[B23-behavsci-15-01246] Greenwald A. G., McGhee D. E., Schwartz J. L. K. (1998). Measuring individual differences in implicit cognition: The implicit association test. Journal of Personality and Social Psychology.

[B24-behavsci-15-01246] Haidt J. (2000). The Positive emotion of elevation. Prevention & Treatment.

[B25-behavsci-15-01246] Haidt J. (2001). The emotional dog and its rational tail: A social intuitionist approach to moral judgment. Psychological Review.

[B26-behavsci-15-01246] Haidt J., Keyes C. L. M., Haidt J. (2003). Elevation and the positive psychology of morality. Flourishing: Positive psychology and the life well-lived.

[B27-behavsci-15-01246] He N., Zhu Y. L. (2016). Self-love and other-love: Research on the relationships among narcissism, empathy and implicit altruism. Acta Psychologica Sinica.

[B28-behavsci-15-01246] Hepach R., Tomasello M. (2020). Young children show positive emotions when seeing someone get the help they deserve. Cognitive Development.

[B29-behavsci-15-01246] Hepach R., Vaish A., Tomasello M. (2012). Young children are intrinsically motivated to see others helped. Psychological Science.

[B30-behavsci-15-01246] Jiang D., Wang X. R., Fu L., Zhou R. L. (2008). A study on implicit altruistic behavior. Psychological Science.

[B31-behavsci-15-01246] Krettenauer T., Campbell S., Hertz S. (2013). Moral emotions and the development of the moral self in childhood. European Journal of Developmental Psychology.

[B32-behavsci-15-01246] Lai C. K., Haidt J., Nosek B. A. (2014). Moral elevation reduces prejudice against gay men. Cognition and Emotion.

[B33-behavsci-15-01246] Liu H. Y. (2012). The relationship between the status of being bullied, prosocial value orientation and interpersonal satisfaction of pupils in primary school. Master’s thesis.

[B34-behavsci-15-01246] Mastromatteo L. Y., Girardi P., Miller J. G., Scrimin S. (2024). Moderate cardiac vagal tone is associated with more cooperation in children. International Journal of Psychophysiology.

[B35-behavsci-15-01246] McGuire A. P., Hayden C., Frankfurt S. B., Kurz A. S., Anderson A. R., Howard B. A. N., Szabo Y. Z. (2020). Social engagement early in the US COVID-19 crisis: Exploring social support and prosocial behavior between those with and without depression or anxiety in an online sample. Journal of Social and Clinical Psychology.

[B36-behavsci-15-01246] Miller J. G., Kahle S., Hastings P. D. (2017). Moderate baseline vagal tone predicts greater prosociality in children. Developmental Psychology.

[B37-behavsci-15-01246] Monroe A. (2020). Moral elevation: Indications of functional integration with welfare trade-off calibration and estimation mechanisms. Evolution and Human Behavior.

[B38-behavsci-15-01246] Pergantis P., Bamicha V., Doulou A., Christou A. I., Bardis N., Skianis C., Drigas A. (2025). Assistive and emerging technologies to detect and reduce neurophysiological stress and anxiety in children and adolescents with autism and sensory processing disorders: A systematic review. Technologies.

[B39-behavsci-15-01246] Piaget J. (1932). The moral judgment of the child.

[B40-behavsci-15-01246] Piaget J. (1964). Part I: Cognitive development in children: Piaget development and learning. Journal of Research in Science Teaching.

[B41-behavsci-15-01246] Piper W. T., Saslow L. R., Saturn S. R. (2015). Autonomic and prefrontal events during moral elevation. Biological Psychology.

[B42-behavsci-15-01246] Porges S. W. (1995). Orienting in a defensive world: Mammalian modifications of our evolutionary heritage. A polyvagal theory. Psychophysiology.

[B43-behavsci-15-01246] Porges S. W. (2007). The polyvagal perspective. Biological Psychology.

[B44-behavsci-15-01246] Porges S. W., Furman S. A. (2011). The early development of the autonomic nervous system provides a neural platform for social behaviour: A polyvagal perspective. Infant and Child Development.

[B45-behavsci-15-01246] Schnall S., Roper J., Fessler D. M. T. (2010). Elevation leads to altruistic behavior. Psychological Science.

[B46-behavsci-15-01246] Schwartz S. H., Howard J. A. (1984). Internalized values as motivators of altruism. Development and maintenance of prosocial behavior.

[B47-behavsci-15-01246] Smetana J. G. (2006). Social-cognitive domain theory: Consistencies and variations in children’s moral and social judgments. Handbook of moral development.

[B48-behavsci-15-01246] Sowell E. R., Thompson P. M., Leonard C. M., Welcome S. E., Kan E., Toga A. W. (2004). Longitudinal mapping of cortical thickness and brain growth in normal children. Journal of Neuroscience.

[B49-behavsci-15-01246] Tangney J. P., Stuewig J., Mashek D. J. (2007). Moral emotions and moral behavior. Annual Review of Psychology.

[B50-behavsci-15-01246] Vecchio G. M., Zava F., Cattelino E., Zuffianò A., Pallini S. (2023). Children’s prosocial and aggressive behaviors: The role of emotion regulation and sympathy. Journal of Applied Developmental Psychology.

[B51-behavsci-15-01246] Xu P. F., Huang Y. X., Luo Y. J. (2010). Establishment and assessment of a native Chinese affective video system. Chinese Mental Health Journal.

[B52-behavsci-15-01246] Yang L. (2012). The development of emotional competence in lower primary school students and its relationship with prosocial behavior. Master’s thesis.

[B53-behavsci-15-01246] Yarritu I., Méndez I., Idoiaga-Mondragón N. (2025). Analysis of the moral elevation experience in adolescents. Current Psychology.

[B54-behavsci-15-01246] Zelazo P. D., Carlson S. M. (2012). Hot and cool executive function in childhood and adolescence: Development and plasticity. Child Development Perspectives.

